# Cognitive characteristics of older Japanese drivers

**DOI:** 10.1186/1880-6805-31-2

**Published:** 2012-02-29

**Authors:** Indri H Susilowati, Akira Yasukouchi

**Affiliations:** 1 Department of Human Science Design, Graduate School of Design, Kyushu University, Shiobaru 4-9-1 Minami ku, Fukuoka 815-8540, Japan; 2 Department of Occupational Health and Safety, Faculty of Public Health, Universitas Indonesia, C Building 1st Floor, Kampus UI Depok 16424, Indonesia

**Keywords:** older driver, cognitive characteristic, Trail Making Test A and B, driver stress inventory, driver coping questionnaire

## Abstract

**Background:**

Some causes of accidents among older drivers are: not paying attention to traffic signals; missing stop lines; and having to deal with and misjudging emergency situations. These causes of accidents reveal problems with attention and cognition. Such incidents are also related to driver perception and stress-coping mechanisms. It is important to examine the relation of stress reactions to attention and cognition as a factor influencing the causes of accidents commonly involving older drivers.

**Finding:**

Subjects were 10 young drivers (23.3 ± 3.33 years) and 25 older drivers divided into two groups (older1 [60 to 65 years] and older2 [> 65 years]). This study revealed the correlation within driver stress inventory and driver coping questionnaires parameters was observed only in older drivers. They also needed a longer response time for Trail Making Test A and B. The factors affected the attention and cognition of older drivers by age but not driving experience itself, and coping parameters such as emotion focus, reappraisal, and avoidance were not included as stress inventory parameters. Being prone to fatigue was less for younger drivers than older drivers. Because they have shorter distances, shorter drive times, and no need for expressways, older drivers also had a significantly lower risk of thrill-seeking behaviour and more patience.

**Conclusion:**

The intervention addressing their attention skills, aggressive feelings, and emotion focus should be considered. The technological improvements in cars will make older drivers feel safer and make driving easier which might lower the attention paid to the road, and regular driving training might be needed to assess and enhance their safety.

## Background

Data from the Japan Automobile Manufacturers Association (JAMA) in 2003 indicated that the ratio of older drivers (aged > 75 years) was twice that of young drivers (aged 16 to 24 years). In 2010, the number of older drivers remains high, with 8.9 million older drivers aged 65 to 74 years and 3.6 million aged > 75 years. JAMA predicts these numbers will increase further, especially for drivers in the latter age category.

Older drivers often have some visual, cognitive, and motor skill limitations but they still need to drive in daily life for health maintenance, social, and leisure reasons. This is especially true in suburban and rural areas where public transportation is limited. Older drivers should remain active and independent in their daily life. Because it is not good for their physical and mental health to stop them from driving, it is vital to ensure that they can drive safely. It is very essential to improve the quality of life in older drivers. Along this line, it is important to see the aging effect on adaptability to driving and the road environment in the field of physiological anthropology.

**Figure 1:**
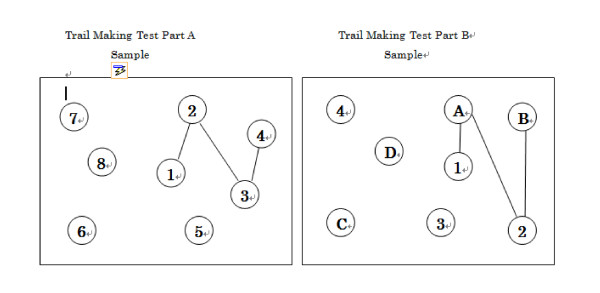
**Sample of TMT A and B test sheet**.

Based on crash data published by JAMA, over 50% of crashes on the roads involving older drivers occurred with automobiles. As an example, 189 cases of road accidents were caused by older drivers during 2009 in Fukuoka prefecture. Some of the common causes of accidents were: not paying attention to traffic signals; missing stop lines; having to deal with emergency situations; and misjudging the speed of oncoming vehicles at crossroads. These causes would seem to reflect problems with attention and cognition. The Trail Making Test (TMT) is one of the most common cognitive tests used to consider visual search and attention, mental flexibility, motor function, and executive function [1]. The TMT consists of two parts, A and B (Figure 1). The task in TMT B is more difficult than in TMT A. TMT B also differs in factors of motor control, perceptual selection demand, and cognitive complexity.

Among older drivers, who have much experience of driving, the cognitive functions necessary for driving would generally be sufficient for routine car trips, thus giving them a perception that their driving behaviour was safe. Such perception can make them overly confident, particularly on streets they are familiar with. However, with certain road conditions or in emergency situations, older drivers who perceive themselves as safe drivers may well become involved in a road accident. Such incidents are related to driver perception and stress-coping mechanisms, factors which can be measured using the driver stress inventory (DSI) questionnaire and the driver coping questionnaire (DCQ), respectively. The DSI is an extension of an earlier assessment of stress, the driver behaviour inventory (DBI), which aimed to measure vulnerability to commonplace stress reactions while driving, such as frustration, anxiety, and boredom [2]. The DCQ determines how the driver handles stress while driving. The coping mechanism employed by drivers is critical for maintaining safety while driving. Other research [3] found significant correlations between DCQ parameters with self-report violations, preferred speed, and inadvertent error.

The study that used the DBI to measure driver stress among Japanese drivers aged 18 to 77 years (*n *= 510) found that several DBI factors predicted accident involvement and convictions for driving offences, with an aggressive driving dimension being the strongest predictor across the criteria [4]. As mentioned above, the DSI is an extension of the DBI which aims at measuring vulnerability to commonplace stress reactions while driving. It is important, therefore, to conduct further research on driver stress among Japanese drivers using the DSI and to clarify their stress coping process using the DCQ. Furthermore, it is important to examine the relation of stress reactions to attention and cognition (as determined by the TMT A and B) as a factor influencing the causes of accident commonly involving older drivers.

Accordingly, the aims of the present study were three-fold: to determine the attention and cognitive characteristics of young and older drivers evaluated by the TMT A and B; to examine how relevant TMT A and B is with the DSI and DCQ; and to clarify whether age and driving experience influences scores on the DSI and DCQ and TMT A and B.

## Methods

Subjects were 10 young drivers and 25 older drivers. Older drivers were recruited from a silver manpower centre (a job creation project for those aged over 60 years in Japan). The subjects (14 males, 11 females) were divided into two age groups: older group 1 drivers for those aged 60 to 65 years (11 persons, mean age 61.9 ± 1.70 years) and older group 2 drivers for those aged over 65 years (14 persons, mean age 69.5 ± 3.01 years). The young drivers were all university students (5 males, 5 females) aged 21 to 32 years (23.3 ± 3.33 years).

Cognitive skills were assessed by the attention test of the TMT A and B, and driver risk perception (DRP) was assessed by the DSI and DCQ. For TMT A, subjects were required to make a line connecting consecutive number targets, while for TMT B subjects connected numbers alternating with letters (for example, 1-A-2-B-3-C and so on). The time taken to complete the test was used as the primary performance metric.

The DSI parameters consist of aggression, dislike of driving, hazard monitoring, fatigue proneness, and thrill-seeking. Dislike of driving and aggression are broad stress syndromes associated with differing cognitive reactions to driving. Aggression is characterized by negative appraisals of other drivers and confrontive coping expressed through intimidation or competition with other drivers; these cognitive processes tend to generate, first, feelings of anger and, second, dangerous driving behaviors which reduce safety. In contrast, dislike of driving is associated with negative self-appraisal and use of emotion-focused coping strategies such as self-blame, which are cognitions that generate negative mood states and worries which tend to interfere with task performance. The third parameter is hazard monitoring, which is related with alertness on the DBI but has a narrower scope, and may be related to a specific style of task-focused coping. Fatigue proneness, which measures the extent to which a driver is prone to driver fatigue after prolonged driving, is related to driver error and lower driving speeds, and is the single strongest predictor of task-induced fatigue symptoms while driving. Thrill-seeking is associated with hazardous behavior that facilitates the sensation and thrill of driving, and is linked to risky driving and increased accident involvement [5].

The DCQ parameters comprise confrontive coping, task focus, emotion focus, reappraisal, and avoidance. Confrontive coping relates to several behaviours that can be considered dangerous and task focus is associated with behaviours likely to enhance safety. Emotion focus is an ineffective strategy in which a driver criticizes and blames him or herself as a driver, an approach that can lead to driver distraction and contribute to the risk of being involved in a collision. Reappraisal involves trying to gain something worthwhile from driving and a feeling of becoming a more experienced driver. Lastly, avoidance is comprised of several types of behaviour in which drivers attempt to ignore the actual situation by focusing on other situations, behavior which often occurs in traffic jams and in accident situations.

Cognitive skills and DRP were both measured by paper-based tests. DSI and DCQ scores were each categorized into three levels of risk (low, moderate, and high) based on the 33rd and 66th percentiles for each parameter. The comparisons within and between the DSI and DCQ were made using the raw score for each parameter. Statistical analysis was performed using SPSS software version 17.0. An ANOVA was used for the comparative analysis, and bivariate, and multiple regression analysis was used to test for correlations.

## Results

**Figure 2:**
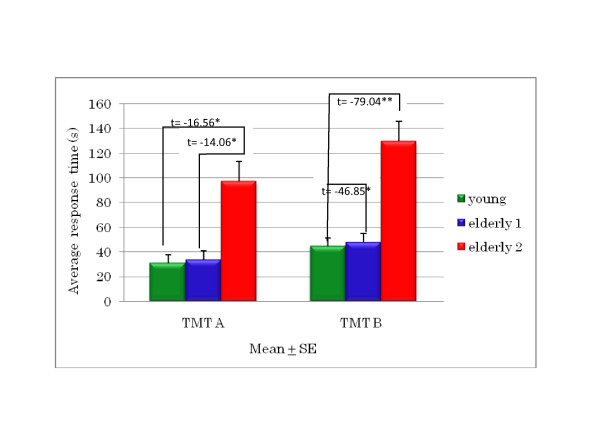
**Trail Making Test A and B results for older and young drivers**.

The results of the TMT A and B revealed that older drivers needed more time to complete the tasks than young drivers. A longer time was needed to complete the TMT B than the TMT A (Figure 2). The comparison shows significant differences for the TMT A between young drivers with older group 2, and between older groups 1 and 2. For TMT B, there was a significant difference found between young drivers and both elderly groups 1 and 2.

Driving experience was measured by how long the subject had held a driver's licence (from the first time they got their driver's licence). Older drivers had held their driver's licence for between 14 and 52 years. On the other hand, young drivers had held their licences for between 1 and 12 years. There is no other significant difference of driving experience between the young and older drivers.

**Figure 3:**
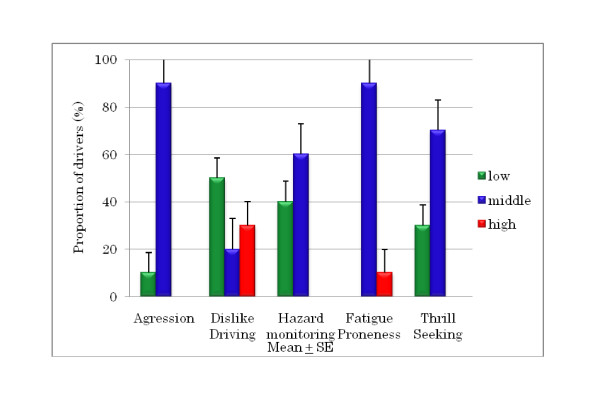
**Proportion of risk for DSI parameters in young drivers**.

**Figure 4:**
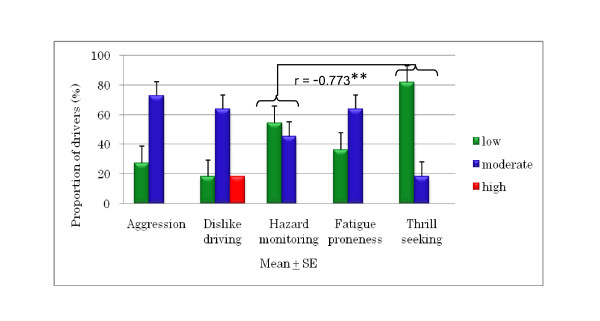
**Correlations of DSI parameters and risk in older group 1 drivers (aged 60 to 65 years)**.

**Figure 5:**
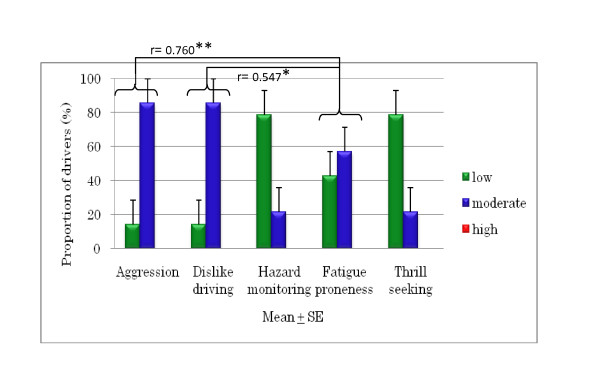
**Correlations of DSI parameters and risk in older group 2 drivers (aged > 65 years)**.

**Figure 6:**
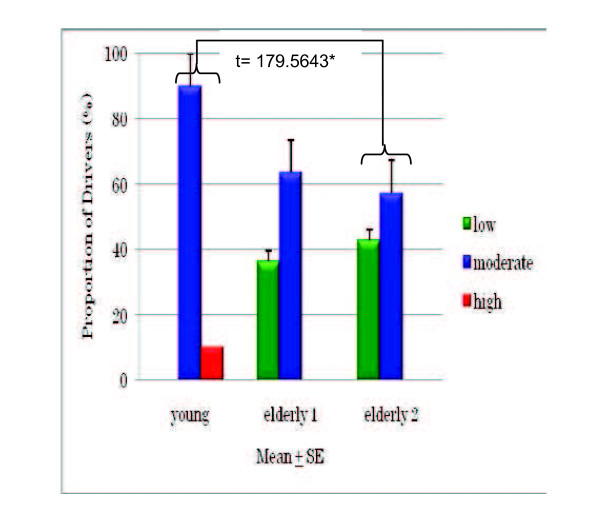
**Comparison of fatigue proneness between young and older drivers**.

**Figure 7:**
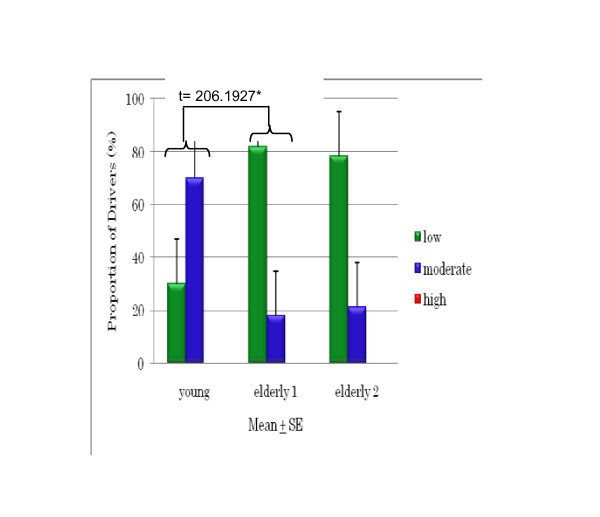
**Comparison of thrill-seeking between young and older drivers**.

The DSI results for young and older drivers (Figures 3, 4, and 5) show that 90% of the young drivers had a moderate risk of aggression, similar to that of the older group 2 drivers. For young and older group 1 drivers, 30% and approximately 20%, respectively, had a high risk of dislike of driving. In terms of hazard monitoring, 60% of young drivers had a moderate risk, compared to both older driver groups who mostly had a low risk. Young drivers had a 90% moderate risk and a 10% high risk of fatigue proneness, and around 60% of both older driver groups had a moderate risk, but none had a high risk. Among young drivers, 70% showed a moderate risk for thrill-seeking, whereas 80% of both the older driver groups had a low risk. Significant differences were found between older group 2 drivers and young drivers for fatigue proneness and between older group 1 drivers and young drivers for thrill-seeking (Figures 6 and 7).

In terms of the DSI parameters, a significant difference was found between hazard monitoring and thrill-seeking among the older group 1 drivers, revealing that when hazard monitoring was high, thrill-seeking was low. Significant differences were also seen between agression and fatigue proneness and between dislike of driving and fatigue proneness among older group 2 drivers, indicating that when aggression and dislike of driving were high, fatigue proneness was also high.

**Figure 8:**
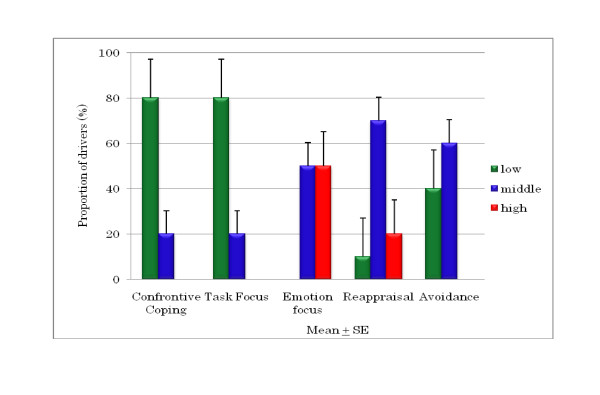
**Proportion of risk for DCQ parameters in young drivers**.

**Figure 9:**
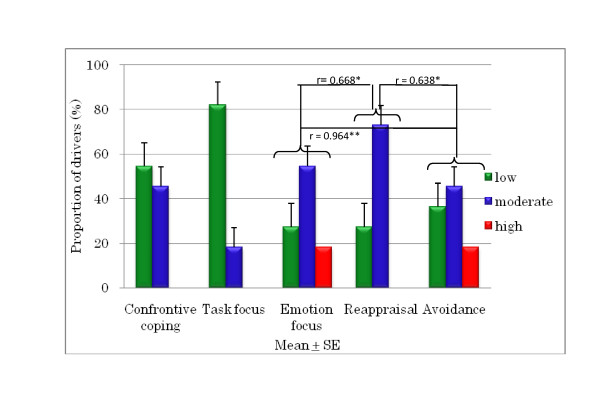
**Correlations between DCQ parameters and risk in older group 1 drivers (aged 60 to 65 years)**.

**Figure 10:**
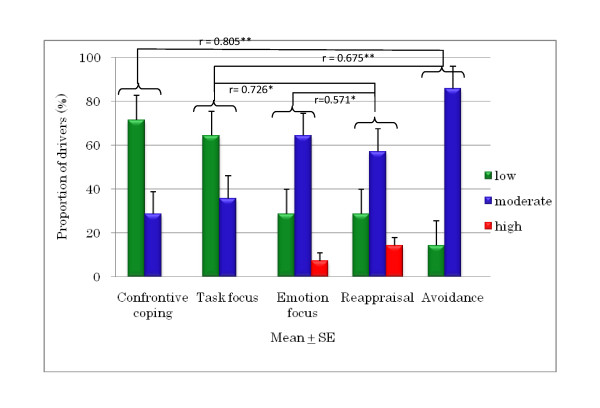
**Correlations between DCQ parameters and risk in older group 2 drivers (aged > 65 years)**.

The DCQ results are shown in Figures 8, 9, and 10. It was found that 80% of young drivers had a low risk for confrontive coping and task focus; both of the older driver groups similarly had predominately low corresponding risks. Among young drivers, 50% had a high risk of emotion focus, whereas most of the older group 1 and 2 drivers had a moderate risk, although around 20% and 7% of the older groups, respectively, had a high risk. For young drivers, 70% had a moderate risk and 20% had a high risk of reappraisal, compared to no high risk among older group 1 drivers; however, around 15% of older group 2 drivers were at high risk of reappraisal. A moderate risk of avoidance was seen in 60% of young drivers and 86% of older group 2 drivers, compared with only 45% of older group 1 drivers. However, there were no significant differences in DCQ items between young drivers and older group 1 and 2 drivers. There was a significant correlation for each of the older driver groups: for the older group 1 drivers, emotion focus was correlated with appraisal and avoidance, meaning that when emotion focus was high, reappraisal and avoidance were similarly high; and for the older group 2 drivers, avoidance had a positive correlation with confrontive coping and task focus, and task focus and emotion focus had a positive correlation with reappraisal.

Multiple regression analysis revealed significant correlations only among the older drivers for the TMT B with age and DCQ parameters like emotion, reappraisal, and avoidance. The coefficient of multiple determinations was 0.693; therefore, about 70% of the variation in the TMT B can be explained by DCQ parameters (emotion, reappraisal, and avoidance) and age. The interpretation of the model is

TMTB=93.147+0.092(emotion)-0.186(reappraisal)-0.96(avoidance)+5.096(age)

This means that every 1 point scored on the TMT B by elderly drivers can be defined by a DCQ score of 0.092 for emotion, -0.186 for reappraisal, -0.96 for avoidance, and 5.096 for age with a prediction value of around.

## Discussion

Attention is the cognitive process of selectively concentrating on one aspect of the environment while ignoring other aspects. Attention has also been referred to as the allocation of processing resources. While driving, it is vital that the driver focuses his or her attention on the road, traffic signs, and other vehicles. TMT is one of the important tests to evaluate attention/cognitive skill. It is already known if TMT is used as a parameter reflex to cognitive.

This study's findings revealed that TMT A and B performance differs significantly between the older driver groups and young drivers (Figure 2). The increasing of age seems to increase the time taken to complete the TMT. This finding indicates that older drivers show decreased attention/cognition, and it is necessary to support the lower level of attention/cognition with technology and/or infrastructure in the traffic environment so that they can improve their driving performance. It is also demonstrated that age influences TMT scores, which is in good agreement with those of Hamdan and Hamdan [6]. Besides that, Tombaugh [7] also found that there was no significant difference of TMT A and B between males and females aged 18 to 89 years. However, this research did not find a significant difference between males and females, for both TMT A and B. Because of that, the data are only divided by age.

From the multiple regression results, it was suggested that attention and cognition (TMT B) among older drivers is correlated with age and with driver coping strategy as evaluated by the DCQ, and particularly emotion focus, reappraisal, and avoidance. The DSI and driving experience did not independently correlate with attention or cognition (TMT A and B). This finding indicates that the time older drivers need to complete the TMT B can be predicted by their score on the DCQ (emotion focus, reappraisal, and avoidance) and age with a prediction value of about 70%. Thus, older drivers take longer to complete the TMT B (reflecting lower attention and cognition abilities), which was affected by age and the DCQ parameters. This correlation of the TMT B with age and DCQ parameters might explain older drivers making oversights at traffic signals or stop lines that cause the kinds of accidents commonly involving older drivers.

According to the analysis results for the DSI, correlations between DSI parameters were found only for older drivers. Fatigue proneness showed significant correlations with aggression and dislike of driving in older drivers aged over 65 years (Figure 5). Fatigue proneness is related to driver error and driving at lower speeds, and is the single strongest predictor of task-induced fatigue symptoms while driving. It is suggested that elderly drivers become easily fatigued when they become angry and blame themselves while driving. To avoid unsafe driving among older people, it is important for them to maintain a stable emotional state, especially in terms of emotions which can clearly have a negative effect on driving skills.

An unexpected result was that young drivers had significantly higher fatigue proneness scores than older drivers aged over 65 years (Figure 6). In a study carried out by Fukuoka prefecture with 10,856 elderly drivers in 35 prefectures across Japan, around 75% of older people drive within 1 h per one driving for their daily activities, and around 90% drive on expressways a few or no times per year (unpublished work).

These facts might account for the lower scores of fatigue proneness among the older drivers in the present study. However, professional drivers (for example, drivers of taxis, minibuses, and heavy vehicles) will almost certainly feel more fatigue irrespective of their age [8].

Research [4] found that young Japanese drivers were more aggressive than older drivers and that those male drivers were also more aggressive than female ones. However, no such difference between young and older drivers was found in the present study. In studies of drivers in the UK, drivers across all age groups were found to show a tendency for aggression, appraising other drivers as hostile and coping confrontationally, a style of coping linked to both anger and dangerous driving [3,9,10]. These studies also demonstrated that undesirable driving behaviour is linked to coping strategies, such as emotion-focused coping and avoidance. Our study also found the same result, that the DSI parameters of aggression and thrill-seeking were correlated with the DCQ parameter of confrontive coping among older drivers aged 60 to 65 years. These drivers also showed correlations between the DCQ parameters of emotion focus and reappraisal and between confrontive coping and avoidance. Older Japanese drivers aged over 65 years might tend to blame themselves and ignore the current situation. However, they showed a tendency to cope with negative perceptions of emotion focus by taking into account many positive things they have experienced in their many years of driving. A study by Kontogiannis [11] on the coping strategies of Greek drivers (*n *= 714, young to middle-aged) found that although drivers who scored highly for aggression also had higher rates of mistakes and violations, this association was not linked to emotion focus but to confrontive coping. Our results for older drivers aged 60 to 65 years are in good agreement, although their sample did not include older drivers aged over 60 years.

Thrill-seeking is associated with hazardous behavior; it facilitates the sensation and thrill of driving, and is linked to risky driving and increased accident involvement. Sometimes young drivers like to frighten themselves a little while driving and get a thrill out of driving fast. The research [8] found that thrill-seeking behavior was related with speeding on in-city roads among young drivers in Turkey. Our study revealed that older drivers had a significantly lower risk of thrill-seeking behavior compared to young drivers (Figure 7), indicating that older drivers are more patient when driving. Our study also found a significant negative correlation between the DSI parameters of thrill-seeking and hazard monitoring in older drivers aged 60 to 65 years (Figure 4), revealing that older drivers in Japan do engage in hazard monitoring and low-level thrill-seeking.

To summarize, this study revealed the following characteristics of attention and cognition and their related factors among older Japanese drivers. The intimate relationship between DRP evaluated on the DSI and driver coping strategy evaluated on the DCQ was observed only in older drivers, not in young drivers. Similar to the findings of other studies that age affects attention and/or cognition, this study revealed that older drivers need longer to respond to the TMT A and B than young drivers. It was suggested that the factors affecting the attention and cognition of older drivers could well be age, but not driving experience, and the DCQ parameters including emotion focus, reappraisal, and avoidance, but not the DSI parameters. When older drivers aged over 65 years feel aggressive, angry, and blame themselves while driving, they might feel fatigue. However, the fatigue proneness of older drivers was less than that of young drivers. This might be caused by their routine driving behaviours, that is, driving short distances and not using the expressway. Moreover, older drivers also had a significantly lower risk of thrill-seeking behaviour and were more patient when driving.

In regard to the cognitive characteristics of older Japanese drivers, intervention addressing their attention skills, aggressive feelings, and emotion focus should be considered. It is possible that technological improvements to cars make older drivers feel safe and that driving is easy which might lower their attention to the road, and therefore regular driving training might be needed to assess and enhance their safety on the roads.

## Abbreviations

TMT A and B: Trail Making Test A and B; DRP: driver risk perception; DBI: driver behaviour inventory; DSI: driver stress inventory; DCQ: driver coping questionnaire; JAMA: Japan Automobile Manufacturers Association.

## Competing interests

The authors declare that they have no competing interests.

## Authors' contributions

IHS carried out in the design of the study and performed the statistical analysis, interpretation of data, and drafting the manuscript or revising it. AY participated in the concept and design of the study, the sequence alignment, and checked the manuscript. All authors read and approved the final manuscript.
